# What is the effect of MRI with targeted biopsies on the rate of patients discontinuing active surveillance? A reflection of the use of MRI in the PRIAS study

**DOI:** 10.1038/s41391-021-00343-2

**Published:** 2021-04-08

**Authors:** H. B. Luiting, S. Remmers, R. Valdagni, E. R. Boevé, F. Staerman, J. Rueb, D. M. Somford, T. Pickles, A. Rannikko, M. J. Roobol, the PRIAS consortium

**Affiliations:** 1 Department of Urology, Erasmus University Medical Centre Cancer Institute, Rotterdam, The Netherlands; 2 Department of Oncology and Haemato-oncology, Università degli Studi di Milano. Radiation Oncology 1, Prostate Cancer Program, Fondazione IRCCS Istituto Nazionale dei Tumori, Milan, Italy; 3 Department of Urology, Sint Franciscus Hospital, Rotterdam, The Netherlands; 4 Department of Urology, Polycliniques REIMS-BEZANNES, Reims, France; 5 Department of Urology, Marien Hospital Herne, Herne, Germany; 6 Department of Urology, Canisius Wilhelmina Hospital, Nijmegen, The Netherlands; 7 Department of Radiation Oncology, Vancouver Center, BC Cancer, Vancouver, BC Canada; 8 Department of Urology, University of Helsinki and Helsinki University Hospital, Helsinki, Finland; 9 Research Program in Systems Oncology, University of Helsinki, Helsinki, Finland

**Keywords:** Cancer epidemiology, Outcomes research

## Abstract

**Background:**

The reduction of overtreatment by active surveillance (AS) is limited in patients with low-risk prostate cancer (PCa) due to high rates of patients switching to radical treatment. MRI improves biopsy accuracy and could therewith affect inclusion in or continuation of AS. We aim to assess the effect of MRI with target biopsies on the total rate of patients discontinuing AS, and in particular discontinuation due to Grade Group (GG) reclassification.

**Methods:**

Three subpopulations included in the prospective PRIAS study with GG 1 were studied. Group A consists of patients diagnosed before 2009 without MRI before or during AS. Group B consists of patients diagnosed without MRI, but all patients underwent MRI within 6 months after diagnosis. Group C consists of patients who underwent MRI before diagnosis and during follow-up. We used cumulative incidence curves to estimate the rates of discontinuation.

**Results:**

In Group A (*n* = 500), the cumulative probability of discontinuing AS at 2 years is 27.5%; GG reclassification solely accounted for 6.9% of the discontinuation. In Group B (*n* = 351) these numbers are 30.9 and 22.8%, and for Group C (*n* = 435) 24.2 and 13.4%. The three groups were not randomized, however, baseline characteristics are highly comparable.

**Conclusions:**

Performing an MRI before starting AS reduces the cumulative probability of discontinuing AS at 2 years. Performing an MRI after already being on AS increases the cumulative probability of discontinuing AS in comparison to not performing an MRI, especially because of an increase in GG reclassification. These results suggest that the use of MRI could lead to more patients being considered unsuitable for AS. Considering the excellent long-term cancer-specific survival of AS before the MRI era, the increased diagnostic accuracy of MRI could potentially lead to more overtreatment if definitions and treatment options of significant PCa are not adapted.

## Introduction

An active surveillance (AS) strategy after the diagnosis of low-risk prostate cancer (PCa) aims to defer or even avoid radical treatment with its associated side effects. Currently available long-term data show that AS has excellent long-term outcomes with cancer-specific survival rates of more than 98% at 10 years [1–3]. Consequently, AS is now a recommended treatment strategy for low-risk PCa included in different guidelines [4, 5]. The high percentage of patients on AS switching to radical treatment (>35% at 5 years), however, limits the reduction of overtreatment by AS [6, 7].

The Prostate Cancer Research International Active Surveillance (PRIAS) study is a prospective study initiated in 2006 to provide a protocolled follow-up strategy for patients on AS [8]. In 2013, the use of MRI with possible targeted biopsies (TBx) was incorporated in the PRIAS follow-up scheme (MRI side study). The addition of MRI with possible TBx to systematic biopsies (SBx) provides a more accurate prostate sampling, reducing the number of PCa Grade Group (GG) > 1 missed [9, 10]. It is known that Gleason Grade upgrading in the first few years of AS is most likely due to inaccurate sampling of the prostate at inclusion (i.e. GG reclassification) rather than due to the actual progression of PCa [11]. The performance of MRI during or before AS is, therefore, thought to influence the rate of GG reclassification while being on AS. Using the data of the PRIAS study, we assessed the rates and reason of discontinuation of AS in patients without an MRI, with an MRI after diagnosis and an MRI before diagnosis. The aim is to provide insight in two important questions: (1) Does the use of MRI at the time of diagnosis affects the 2-year discontinuation rate? And (2) Does the inclusion of MRI as a follow-up tool affects the GG reclassification rate in patients already on AS?

## Methods

The PRIAS study is a web-based register (www.prias-project.org) in which clinicians from around the world can enter data of their low-risk PCa patients who opted for an AS strategy after diagnosis. The website, based on monitoring data entered in the web application (e.g. PSA or outcome of digital rectal examination (DRE) or prostate biopsy), recommends on an individual level to continue AS or to switch to radical treatment. All patients included in this analysis provided informed consent.

### PRIAS protocol

The criteria for inclusion in the PRIAS study in 2006 were GG ≤ 1, clinical stage ≤T2c, PSA ≤ 10 ng/ml, ≤2 cores positive for PCa, PSA-density ≤0.2 ng/ml/cm^3^, and fitness for curative treatment [12]. Since 2013, patients with >2 cores positive for PCa are allowed in the PRIAS side study in which MRI is used before diagnosis or during follow-up (see Supplementary file Table 1) [12].

The PRIAS study follow-up protocol has been described in detail in earlier publications [6, 12]. In summary, regular PSA measurement and DRE are performed. A repeat biopsy session is recommended 1, 4, 7, and 10 years after inclusion. If the PSA doubling time is below 10 years, a yearly biopsy session is recommended. The in 2013 initiated PRIAS MRI side study recommends performing an MRI with TBx 3 months after inclusion and in combination with the SBx after 1, 4, 7, and 10 years. If an MRI was used before inclusion, the performance of an MRI after 3 months should be omitted (see Supplementary file 1 Table 2). If the PSA doubling time is below 10 years and MRI is available for clinical use, annual MRI is recommended with TBx only if MRI shows progression (i.e. new lesions or increased susceptibility of known lesions e.g. PRECISE score 4 or 5 [13]) instead of yearly biopsy sessions. If a patient switched to radical therapy without evidence of progression based on the follow-up criteria of the PRIAS protocol, or anxiety was not explicitly mentioned as the reason of discontinuation, the event was recorded as discontinuation without progression.

At the initiation of the PRIAS study, a switch to radical treatment was advised if the PSA doubling time was below 3 years, if >2 biopsy cores showed PCa, if GG > 1 was detected or if DRE showed >cT2. In the MRI side study, the recommendations to switch to radical treatment when PSA doubling time was below 3 years was removed. Moreover, if >2 biopsies showed PCa GG1, it was recommended to continue AS [12].

### Study population

We formed three different subpopulations out of the PRIAS database. The first group (Group A) consists of patients included in the PRIAS study before 2009 (i.e. well before the MRI era). The second group (Group B) consists of patients diagnosed and included in the PRIAS study without MRI. All patients in Group B underwent MRI with possible TBx within 6 months after diagnosis. The third group (Group C) consists of PRIAS patients who underwent MRI before diagnosis and during follow-up.

### Statistical analysis

Descriptive statistics were used to describe the patient characteristics at baseline in each group. The reason for discontinuing AS was recorded. Cumulative incidence curves were used to estimate rates of discontinuation. Censoring included men still on AS or lost to follow-up. We chose to focus on the 2-year cumulative incidence of discontinuation because at this time most patients underwent their recommended confirmatory biopsy at 1 year. Statistical analyses were performed with IBM SPSS software version 25 and R version 3.5.1 [14].

## Results

Groups A, B, and C consist of 500, 351, and 435 patients respectively and their median PSA at inclusion was 5.3 (interquartile range (IQR) 3.9–6.7), 6.0 (IQR 4.8–7.8) and 5.8 (IQR 4.6–7.8) ng/ml. In all groups, at least 80% of patients had T1c disease at the time of diagnosis and at least 46% of the patients had only one biopsy core containing PCa. All patients in groups A and B were diagnosed with PCa GG 1 on SBx. In Group C, SBx at diagnosis showed PCa GG 1 in 368 (85%) patients, 42 (10%) patients had negative SBx at diagnosis but PCa GG 1 was detected in TBx solely, and 25 (6%) patients underwent no SBx at diagnosis. Table 1 displays the patient characteristics at baseline.

**Table 1:** Patients characteristics at the time of inclusion in the PRIAS study.

	Group A	Group B	Group C
Total number of patients	500	351	435
Age at diagnosis median (IQR) years	65 (60–70)	65 (60–70)	65 (60–70)
PSA at diagnosis median (IQR) ng/ml	5.3 (3.9–6.7)	6.0 (4.8–7.8)	5.8 (4.6–7.8)
Prostate volume at diagnosis median (IQR) ml	43 (35–57)	43 (35–57)	50 (38–63)
PSA Density at diagnosis median (IQR) ng/ml/ml	0.12 (0.0–16)	0.14 (0.10–0.18)	0.12 (0.09–0.16)
T-stage			
T1c	413 (82.6%)	315 (89.7%)	385 (88.5%)
T2a	80 (16.0%)	32 (9.1%)	43 (9.8%)
T2b	3 (0.6%)	4 (1.1%)	6 (1.4%)
T2c	4 (0.8%)	0 (0%)	1 (0.2%)
Number of systematic biopsy cores taken median (IQR)	9 (7–12)	12 (10–12)	12 (11–14)
Outcome Systematic biopsies			
No prostate cancer	0 (0%)	0 (0%)	67 (15.4%)
GG 1	500 (100%)	351 (100%)	368 (84.6%)
Number of systematic biopsy cores positive for PCa			
0	0 (0%)	0 (0%)	67 (15.4%)
1	357 (71.4%)	176 (50.1%)	202 (46.4%)
2	138 (27.6)	104 (29.6%)	108 (24.8%)
3	2 (0.4%)	38 (10.8%)	34 (7.8%)
4	1 (0.2%)	20 (5.7%)	14 (3.2%)
>4	0 (0%)	12 (3.4%)	10 (2.3%)
Unknown	2 (0.4%)	1 (0.3%)	
MRI outcome			
No lesion			100 (23%)
PIRADS 3			104 (23.9%)
PIRADS 4			186 (42.8%)
PIRADS 5			37 (8.5%)
Missing data			8 (1.8%)

*IQR* interquartile range, *GG* Grade Group, *PCa* prostate cancer.

Median follow-up in groups A, B, and C of patients who did not discontinue their AS strategy was 10.0 (IQR 5.7–11.9), 2.0 (IQR 0.9–3.2), and 1.0(0.4–2.0) years. The cumulative probability of undergoing at least one biopsy procedure in the first 1.5 years on AS was comparable between the three groups, 90.7% (95% confidence interval (CI) 88.1–93.4) for Group A, 89.7% (95% CI 85.7–93.6) for Group B and 95.4% (95% CI 92.4–8.4) for Group C.

**Fig. 1:**
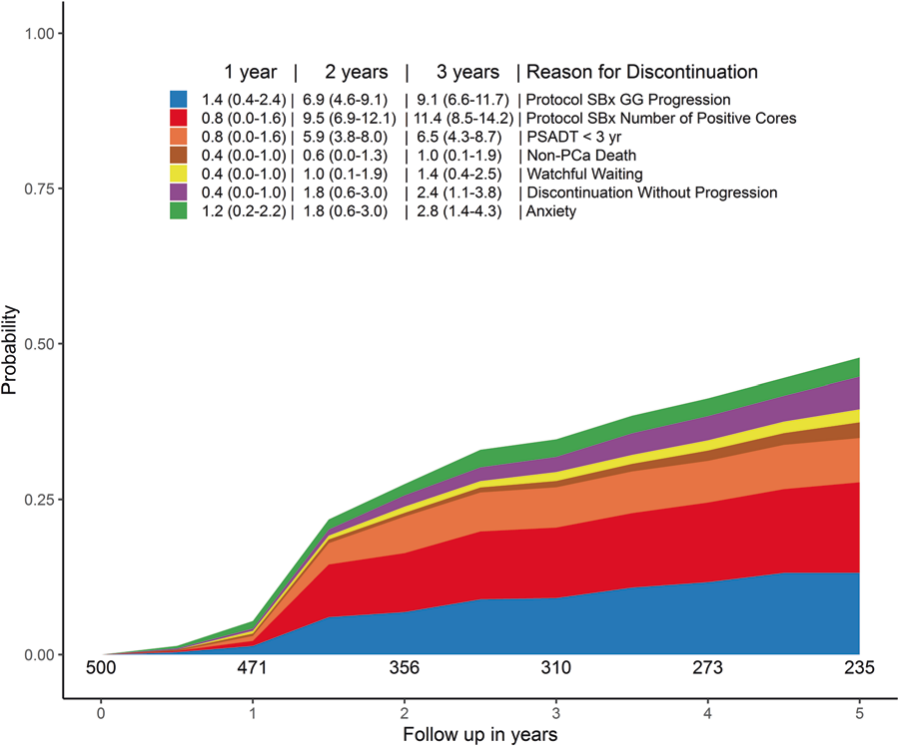
The cumulative incidence rates and reason of discontinuation of the first 500 patients included in the PRIAS study, long before the introduction of MRI with targeted biopsies (i.e. Group A). SBx systematic biopsies, GG Grade Group, PCa prostate cancer.

In Group A, the cumulative probability of discontinuing AS at 2 years is 27.5% (Fig. 1). Of this 27.5%, the probability to discontinue because of disease progression is 22.3%. In more detail, the probability to discontinue AS because of GG reclassification by SBx is 6.9% (95% CI 4.6–9.1), 9.5% (95% CI 6.9–12.1) because of more than 2 cores positive for PCa GG 1, and 5.9% (95% CI 3.8–8.0) because of a PSA doubling time below 3 years. The probability to discontinue AS because of anxiety is 1.8% (95% CI 0.6–3.0).

**Fig. 2:**
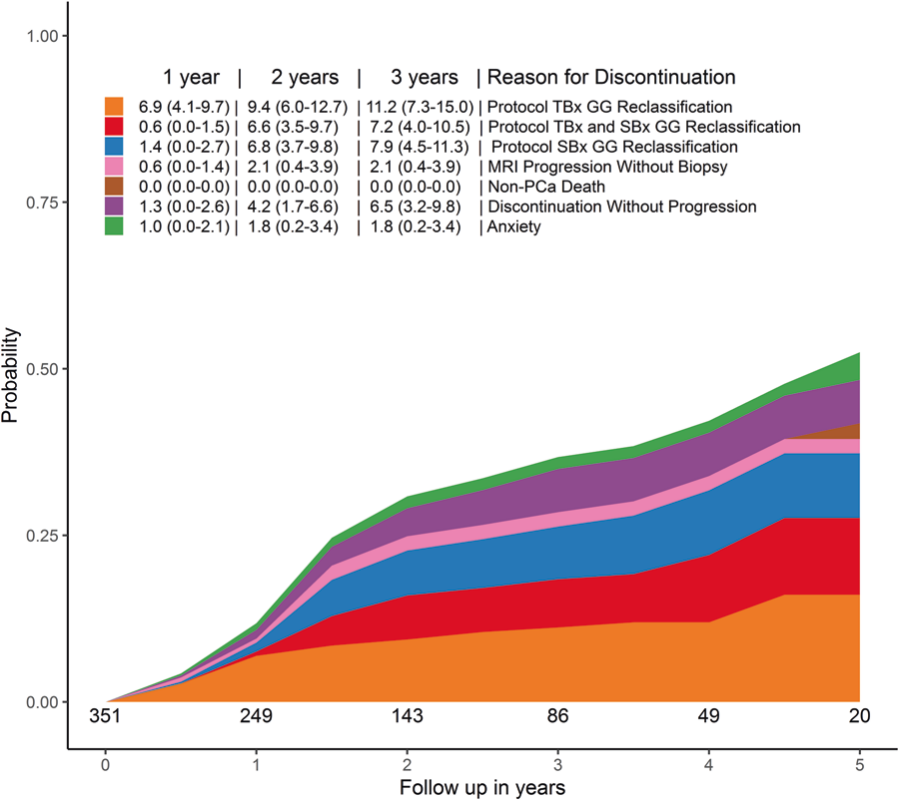
The cumulative incidence rates and reason of discontinuation of patients included in the PRIAS study without upfront MRI but who underwent MRI in the first 6 months after inclusion (i.e. Group B). TBx targeted biopsies, GG Grade Group, SBx systematic biopsies.

In Group B, the cumulative probability of discontinuing AS at 2 years is 30.9% (Fig. 2). Of this 30.9%, the probability to discontinue because of GG reclassification is 22.8%. In more detail, the probability to discontinue AS solely based on the TBx result is 9.4% (95% CI 6.0–12.7), 6.6% (95% CI 3.5–9.7) based on both TBx and SBx result, and 6.8% (95% CI 3.7–9.8) solely based on the SBx result. The probability to discontinue AS because of anxiety is 1.8% (95% CI 0.2–3.4).

**Fig. 3:**
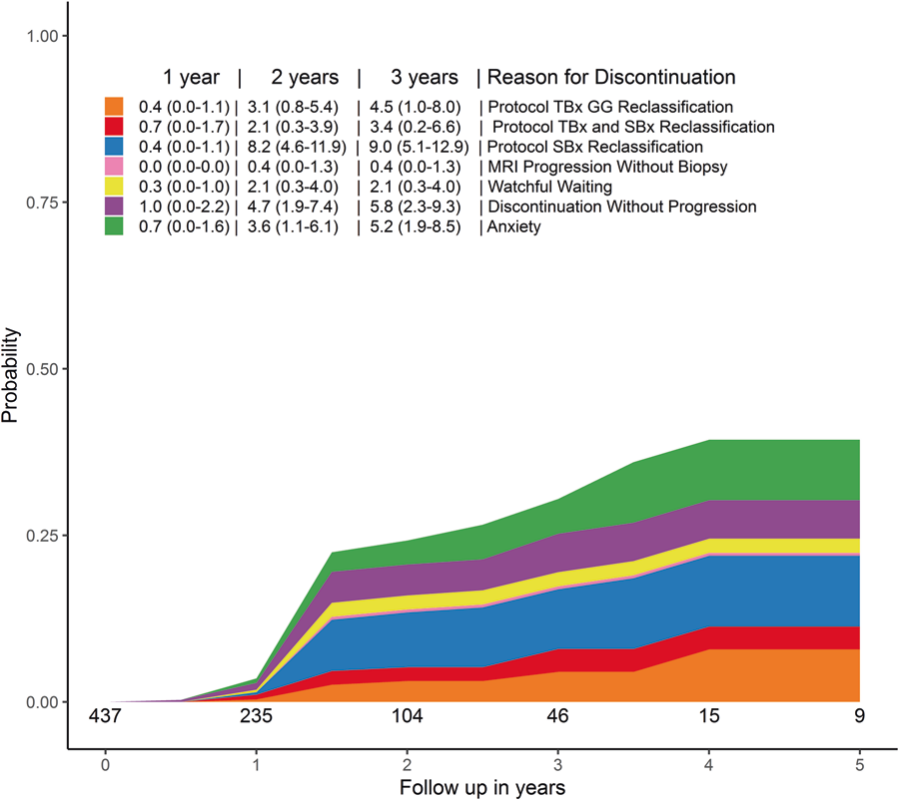
The cumulative incidence rates and reason of discontinuation of patients included in the PRIAS study with upfront (i.e. Group C). TBx targeted biopsies, GG Grade Group, SBx systematic biopsies.

In Group C, the cumulative probability of discontinuing AS at 2 years is 24.2% (Fig. 3). Of this 24.2%, the probability to discontinue because of GG reclassification is 13.4%. In more detail, the probability to discontinue AS solely based on the TBx result is 3.1% (95% CI 0.8–5.4), based on both the TBx and SBx result is 2.1% (95% CI 0.3–3.9), and based on solely the SBx result is 8.2% (95% CI 4.6–11.9). The probability to discontinue AS because of anxiety is 3.6% (95% CI 1.1–6.1%).

## Discussion

In the current analysis, we aimed to provide insight on the effect of MRI on the rate of patients discontinuing AS. To do so, we described the cumulative probability rates of patients discontinuing AS in three subpopulations of patients included in the PRIAS study. The three groups differ with respect to the use of MRI, either no use (Group A), the use of MRI during AS but not before diagnosis (Group B) and lastly the use of MRI before diagnosis and during AS (Group C). Patients who underwent MRI before inclusion in AS (Group C) showed higher probability to continue AS at 2 years in comparison to patients who underwent MRI after diagnosis (Group B). These results are as expected as the performance of MRI with possible TBx in addition to SBx increases the accuracy of prostate biopsy, and as such identifies PCa GG > 1 that would have been missed by the SBx procedure. Looking at the rate of GG reclassification, we observed that in Group A there is a substantially lower 2-year GG reclassification rate (6.9%) as compared to patients in Group B (2-year reclassification rate of 22.8%) and patients in Group C (2-year GG reclassification rate of 13.4%) Considering the similar baseline patient and tumor characteristics in groups A and B (and to a somewhat lesser extend in Group C), we argue that the use of MRI with possible TBx in the AS follow-up schedule is the main reason for these observed higher rates GG reclassification.

Our results showing lower rates of discontinuation in Group C (MRI used both at diagnosis and during AS) as compared to Group B (MRI used in the early phase of AS but not at diagnosis) are in line with the updated results of the ASIST trial, which shows that the performance of MRI with possible TBx early in AS reduces the rate of GG reclassification later during AS [15]. It must, however, be noted that the current data if Group C still show a 2-year probability of 13.4% to reclassify due to GG reclassification despite an MRI-based diagnosis. Recently, Chesnut et al. reported a GG reclassification rate of 32% at 3 years in a cohort of patients on AS who underwent MRI before being included in AS [16]. Although these numbers are not directly comparable to our 2-year data, they support the possibility of incorrect sampling, even with the use of MRI and TBx at diagnosis. In addition, in Group C, the SBx technique was most often (8.2% of the total of 13.4% cases that reclassified = 61%) the sole detection method that identified GG reclassification. This is in accordance with the results of Hamoen et al. showing 12% GG reclassification at 1 year by SBx solely, therefore, strongly supporting the importance of confirmatory systematic biopsies in an AS setting [17].

An AS strategy aims to reduce overtreatment of PCa. The excellent long-term disease-specific survival of patients on AS informs us that for many patients AS is the best treatment strategy. This long-term survival data is based on patients following AS programs based on inclusion and follow-up strategies using SBx only. Our results suggest that the use of MRI with possible TBx during AS is associated with an increase in the probability to discontinue AS due to GG progression. The question now arises whether this increase is needed to allow AS to remain a safe treatment strategy or might in particular increases overtreatment in PCa. While improvement in diagnostic accuracy is something we must embrace, we should, at the same time, challenge the classical definitions of significant disease. Most patients on AS upgraded because of TBx show to have GG2 disease [18]. The group of patients with GG 2 is very heterogeneous and automatically excluding these patients for AS likely leads to overtreatment. Thus, GG criteria for triggering treatment change at the MRI and TBx era needs to be updated accordingly [19–22]. Only then we will avoid that “improvement actually leads to more harm”.

Different guidelines already support the use of AS for selected patients with intermediate-risk PCa [4, 5, 23, 24]. Currently this is not always reflected in actual data; a recent report based on US data shows that less than 5% of patients diagnosed with GG 2 undergo AS [25]. A lower volume of Gleason pattern 4 on biopsy is associated with a more favorable outcome at radical prostatectomy [19]. In addition, the presence of cribriform/intraductal growth patterns (CR/IDC) in PCa shows to be a risk factor for metastases [20–22]. Incorporating CR/IDC grading systems to classify the aggressiveness of PCa detected by biopsies improves the prediction of long-term cancer-specific and metastasis-free survival [20]. As recommended by the EAU guidelines, patients with CR/IDC growth patterns should be excluded for AS [4]. Hence, we argue that if MRI is used to accurately grade PCa and PCa GG 2 without CR/IDC being detected, an AS strategy should be recommended. Finally, it is important to note the rates of discontinuation because of anxiety. We would expect that the increased confidence of prostate sampling by MRI would lead to a drop in the rate of discontinuation because of anxiety. Our data show, however, the opposite. Whatever the reason is, clinicians should be aware that anxiety still contributes to overtreatment in low-risk PCa.

This study is not without limitations. The three subpopulations are included in the PRIAS in different time eras and are not randomized at inclusion. Group B consists of more patients with ≥3 biopsy cores positive for GG 1 at inclusion, maybe reflecting a slightly higher overall risk group. However, still most patients had only 1 or 2 biopsy cores positive. Moreover, more biopsy cores were taken Group B in comparison to Group A. Other baseline patient characteristics are highly comparable (all GG 1, >80% T1c and comparable PSA at diagnosis). We chose to include a cohort from before the MRI era rather than a cohort not undergoing MRI in the MRI era to prevent a selection bias who would undergo MRI. Secondly, the PRIAS study provides data from different centers worldwide. Therefore, quality of MRI assessment, prostate biopsy and pathology evaluation is not equal throughout. However, this is real-life data and as such represents daily clinical practice. Thirdly, the adherence to an AS biopsy protocol is never perfect. Patients who do not undergo a prostate biopsy cannot be reclassified. However, between the three cohorts, the cumulative probability to undergo at least one biopsy procedure in the first 1.5 years on AS is over 90%.

Finally, although our results highlight the importance of confirmatory biopsies, performing biopsies regularly (e.g. annual or every 3 years) results in many unnecessary biopsy sessions. Because prostate biopsies are bothersome and not without risk, these should be avoided as much as reasonably possible [26]. Recently, reporting data from a large cohort of men followed by an MRI-based AS protocol showed a similar rate of patients discontinuing AS while fewer prostate biopsies session were performed in comparison to reported standard AS cohorts [27]. In addition, personalized schedules using clinical data can reduce the number of biopsy sessions without delaying the detection of disease progression [28]. Personalized schedules including MRI outcome are warranted to further reduce the number of biopsy sessions performed during AS [29]. The high cumulative incidence rates of discontinuation in all groups described in this study emphasize that to reduce overtreatment, clinicians should always try to reduce the detection of insignificant PCa at diagnosis, in which MRI can be helpful [9, 29].

## Conclusion

In this study, we assessed the cumulative rate of AS discontinuation in three cohorts who differ in respect to the use of MRI included in the PRIAS study. We showed that the use of MRI with possible TBx before inclusion in AS reduces the probability of discontinuing AS after 2 years. This is most likely due to a more stringent selection at the start of AS. Second, the use of MRI in patients already on AS increases in the cumulative probability to discontinue AS, mostly due to increased GG reclassification. In both cases, considering the current excellent long-term survival of patients on AS diagnosed and (mainly) followed without the use of MRI, it is important to realize that this increased detection of GG reclassification might increase overtreatment. To avoid that a more sensitive method of detection and monitoring will actually result in harm, we suggest that the definition and treatment options of clinically significant PCa in the MRI era should be adapted and implemented as soon as possible.

## Supplementary information

Supplementary table 1

Supplementary table 2

Appendix 1
