# Supplementary material for: What is the effect of MRI with targeted biopsies on the rate of patients discontinuing active surveillance? A reflection of the use of MRI in the PRIAS study
Source: Prostate Cancer Prostatic Dis. 2021 Apr 8;24(4):1048–54. doi: 10.1038/s41391-021-00343-2 (PMC8616762; doi:10.1038/s41391-021-00343-2)
Supplement: Supplementary file 1 — Supplementary table 1 [file 41391_2021_343_MOESM1_ESM.pdf]

Inclusion criteria	Group A	Group B/C
PSA	≤ 10 ng/mL	≤ 10 ng/mL
PSA density	<0,2 ng/mL/cm3	<0,2 ng/mL/cm3
cT stage	<T3	<T3
Grade Group	1	1
Total numbers positive	≤ 2	-
Follow-up criteria		
cT stage	<T3	<T3
Grade Group	1	1
Total numbers positive	≤ 2	-
PSA doubling time	> 3 years	-

Supplementary file Table 1 – The inclusion criteria for patients in group A and group B/C.
