# Supplementary material for: What is the effect of MRI with targeted biopsies on the rate of patients discontinuing active surveillance? A reflection of the use of MRI in the PRIAS study
Source: Prostate Cancer Prostatic Dis. 2021 Apr 8;24(4):1048–54. doi: 10.1038/s41391-021-00343-2 (PMC8616762; doi:10.1038/s41391-021-00343-2)
Supplement: Supplementary file 2 — Supplementary table 2 [file 41391_2021_343_MOESM2_ESM.pdf]

Year	0					2				3		4		5		6		7	
Month	0	3	6	9	12	15	18	21	24	30	36	42	48	54	60	66	72	78	84
PSA	x	x	x	x	x	x	x	x	x	x	x	x	x	x	x	x	x	x	x
DRE	x		x		x		x		x		x		x		x		x		x
Systematic Biopsy	x				x								x						x
Evaluation	x		x		x		x		x		x		x		x		x		x
MRI + possible targeted biopsy		x*			x								x						x

Supplementary file Table 2 – The recommended follow up schedule of the PRIAS study.
In red the addition for group B and C. MRI with targeted biopsies is only recommended for patients without upfront MRI (group B).
