# Supplementary material for: What is the effect of MRI with targeted biopsies on the rate of patients discontinuing active surveillance? A reflection of the use of MRI in the PRIAS study
Source: Prostate Cancer Prostatic Dis. 2021 Apr 8;24(4):1048–54. doi: 10.1038/s41391-021-00343-2 (PMC8616762; doi:10.1038/s41391-021-00343-2)
Supplement: Supplementary file 3 — Appendix 1 [file 41391_2021_343_MOESM3_ESM.docx]

**Appendix 1. List of contributors per medical center.**

| Admiraal de Ruyter Ziekenhuis | Goes/Vlissingen | Netherlands | F.M. Bentvelsen |
| --- | --- | --- | --- |
| Admiraal de Ruyter Ziekenhuis | Goes/Vlissingen | Netherlands | J. Jaspars |
| Admiraal de Ruyter Ziekenhuis | Goes/Vlissingen | Netherlands | E. Planken |
| Aker Sykehus | Oslo | Norway | A. Bendiksby |
| Aker Sykehus | Oslo | Norway | Olav Andreas Hopland |
| Aker Sykehus | Oslo | Norway | V. Berge |
| Akita University Hospital | Akita | Japan | T. Habuchi |
| Albert Schweitzer Ziekenhuis | Dordrecht | Netherlands | R.M. Potjer |
| Albert Schweitzer Ziekenhuis | Dordrecht | Netherlands | R. Raaijmakers |
| Albert Schweitzer Ziekenhuis | Dordrecht | Netherlands | W.M. Stomps |
| Albert Schweitzer Ziekenhuis | Dordrecht | Netherlands | J.J. Vis |
| Albert Schweitzer Ziekenhuis | Dordrecht | Netherlands | P.A. Wertheimer |
| Albert Schweitzer Ziekenhuis | Dordrecht | Netherlands | A.G.M. Zeegers |
| Alysis Zorggroep Arnhem | Arnhem | Netherlands | G. Smits |
| Amphia Ziekenhuis | Breda | Netherlands | P.J. van den Broeke |
| Amphia Ziekenhuis | Breda | Netherlands | D. van der Schoot |
| Amphia Ziekenhuis | Breda | Netherlands | H. Jansen |
| Amphia Ziekenhuis | Breda | Netherlands | J. van Brakel |
| Amphia Ziekenhuis | Breda | Netherlands | K. Zaccai |
| Amphia Ziekenhuis | Breda | Netherlands | M. Tijnagel |
| Amphia Ziekenhuis | Breda | Netherlands | I. van Onna |
| Amphia Ziekenhuis | Breda | Netherlands | E.H.G.M. Oomens |
| Amphia Ziekenhuis | Breda | Netherlands | P.J. Posthumus |
| Amphia Ziekenhuis | Breda | Netherlands | M. Schuit |
| Antoni van Leeuwenhoek Ziekenhuis (NKI-AVL) | Amsterdam | Netherlands | E. van Muilekom |
| Antoni van Leeuwenhoek Ziekenhuis (NKI-AVL) | Amsterdam | Netherlands | H.G. van der Poel |
| BC Cancer Agency | Vancouver | Canada | A. Kahnamelli |
| BC Cancer Agency | Vancouver | Canada | A. Briede |
| BC Cancer Agency | Vancouver | Canada | H. Lau |
| BC Cancer Agency | Vancouver | Canada | T. Pickles |
| Bronovo Ziekenhuis | The Hague | Netherlands | C. Rikken |
| Bronovo Ziekenhuis | The Hague | Netherlands | I. Cordia |
| Bronovo Ziekenhuis | The Hague | Netherlands | M. Kummeling |
| Canisius-Wilhelmina Ziekenhuis | Nijmegen | Netherlands | R. van den Bergh |
| Canisius-Wilhelmina Ziekenhuis | Nijmegen | Netherlands | D. Duijvesz |
| Canisius-Wilhelmina Ziekenhuis | Nijmegen | Netherlands | D.M. Somford |
| Canisius-Wilhelmina Ziekenhuis | Nijmegen | Netherlands | H. Vergunst |
| Catharina Ziekenhuis | Eindhoven | Netherlands | E.L. Koldewijn |
| Catharina Ziekenhuis | Eindhoven | Netherlands | P.E.F. Stijns |
| Catharina Ziekenhuis | Eindhoven | Netherlands | W. Scheepens |
| Centre Hospitalitier Universitaire de Lille | Lille | France | A. Villers |
| Champalimaud Foundation | Lisbon | Portugal | J. Fonseca |
| Charles University | Prague | Czech Republic | O. Capoun |
| Chiba University Hospital | Tokyo | Japan | S. Sakamoto |
| Chu de Bordeaux | Bordeaux | France | R. Gregoir |
| Diaconesses - Croix St Simon Hospital | Paris | France | B. Guillonneau |
| Diakonessenhuis | Utrecht | Netherlands | K. van Dalen |
| Diakonessenhuis | Utrecht | Netherlands | R. Spermon |
| Emco Klinik | Salzburg | Austria | A. Jungwirth |
| Erasmus MC | Rotterdam | Netherlands | W. Boellaard |
| Erasmus MC | Rotterdam | Netherlands | J. Boormans |
| Erasmus MC | Rotterdam | Netherlands | M.B. Busstra |
| Erasmus MC | Rotterdam | Netherlands | B. Weening |
| Erasmus MC | Rotterdam | Netherlands | C.H. Bangma |
| Erasmus MC | Rotterdam | Netherlands | F.H. Drost |
| Erasmus MC | Rotterdam | Netherlands | S. van den Heuvel |
| Erasmus MC | Rotterdam | Netherlands | K. de Mooij |
| Erasmus MC | Rotterdam | Netherlands | W.J. Kirkels |
| Erasmus MC | Rotterdam | Netherlands | L.P. Bokhorst |
| Erasmus MC | Rotterdam | Netherlands | M. Bul |
| Erasmus MC | Rotterdam | Netherlands | M. van Mierlo |
| Erasmus MC | Rotterdam | Netherlands | P.C.M.S. Verhagen |
| Erasmus MC | Rotterdam | Netherlands | M.J. Roobol |
| Erasmus MC | Rotterdam | Netherlands | W. Roobol |
| FCPorto | Porto | Portugal | N. Azevedo |
| Fundaci | Barcelona | Spain | F. Sanguedolce |
| Fundación Hospital de Jove | Gijón | Spain | P.P. Perez |
| G. Fornaroli hospital, Legnano | Milan | Italy | M. Maffezzini |
| Gelderse Vallei Ziekenhuis | Ede | Netherlands | M. Kortleve |
| Gelderse Vallei Ziekenhuis | Ede | Netherlands | A. Viddeleer |
| GSMS - Kumamoto University | Kumamoto | Japan | M. Eto |
| Gunma University | Maebashi City | Japan | K. Suzuki |
| HAGA | The Hague | Netherlands | M. Kiewiet de Jonge |
| HAGA | The Hague | Netherlands | F. Froeling |
| Hamamatsu University Hospital | Higashi-ku | Japan | T. Sugiyama |
| Hara-Sanshin Hospital | Fukuoka | Japan | Shiga |
| Heinrich Heine University of Dusseldorf | Düsseldorf | Germany | C. Arsov |
| Heinrich Heine University of Dusseldorf | Düsseldorf | Germany | D. Caasen-Findeisen |
| Heinrich Heine University of Dusseldorf | Düsseldorf | Germany | R. Rabenalt |
| HELIOS Klinikum Krefeld | Krefeld | Germany | H. Krueger |
| HELIOS Klinikum Krefeld | Krefeld | Germany | J. Schmitges |
| Hiroshima University Hospital | Hiroshima | Japan | J. Teishima |
| Hokkaido University Hospital | Hokkaido | Japan | S. Maruyama |
| Hospital Italiano de Buenos Aires | Buenos Aires | Argentina | C.A. Becerra |
| Hospital Italiano de Buenos Aires | Buenos Aires | Argentina | P.F. Martinez |
| Hospital Italiano de Buenos Aires | Buenos Aires | Argentina | W. Villamil |
| Hospital Universitari Mutua Terrassa | Terrassa | Spain | J. M. Caballero Giné |
| Hospital Universitari Mutua Terrassa | Terrassa | Spain | R. Bastus |
| Hospital Universitario Marques de Valdecilla | Santander | Spain | E.R. Barselo |
| Hospital Universitario San Agustin | Aviles | Spain |  |
| Hospital Virgen del Camino | Pamplona | Spain | M. Montesino |
| HYKS | Helsinki | Finland | A.S. Rannikko |
| IJsselland Ziekenhuis | Capelle aan den IJssel | Netherlands | B. Rikken |
| Iwate university | Morioka | Japan | W. Obara |
| Jeroen Bosch Ziekenhuis | 's-Hertogenbosch | Netherlands | H.P. Beerlage |
| Jeroen Bosch Ziekenhuis | 's-Hertogenbosch | Netherlands | J.R. Oddens |
| Jeroen Bosch Ziekenhuis | 's-Hertogenbosch | Netherlands | R.A. Schipper |
| Jikei Medical University Hospital | Tokyo | Japan | H. Sasaki |
| Kagawa University | Takamatsu | Japan | Y. Kakehi |
| Kansai Medical University Hospital | Osaka | Japan | H. Kinoshita |
| Klinik für Urologie Nagold | Nagold | Germany | A. Gayer |
| Klinik für Urologie Nagold | Nagold | Germany | M. Schiffer |
| Klinikum Aschaffenburg-Alzenau | Aschaffenburg | Germany | J. Winkle |
| Kocaeli University Faculty of Medicine | Kocaeli | Turkey | T.A. Ozkan |
| Krankenhaus der Barmherzigen Brüder | Trier | Germany | N. Schmeller |
| Kuopio University Hospital | Kuopio | Finland | S. Aaltomaa |
| Kurashiki Central Hospital | Kurashiki | Japan | A. Terai |
| Kuusankoski Hospital | Kouvola | Finland | M. Multanen |
| Kyorin University | Tokyo | Japan | Kyorin |
| Kyoto Prefectual University of Medicine | Kyoto | New Zealand | O. Ukimura |
| Kyoto University Hospital | Kyoto | Japan | T. Inoue |
| Kyushu University Hospital | Fukuoka | Japan | A. Yokomizo |
| La Fe University Hospital | Valencia | Spain | C.D. Vera Donoso |
| La Fe University Hospital | Valencia | Spain | J.M. Alapont-Alacreu |
| La Fe University Hospital | Valencia | Spain | M. Martinez-Sarmiento |
| La Paz University Hospital | Madrid | Spain | C. de Castro |
| LUMC | Leiden | Netherlands | M. Roeleveld |
| LUMC | Leiden | Netherlands | R. Pelger |
| Marien Hospital Herne | Herne | Germany | A.M. Reicherz |
| Marien Hospital Herne | Herne | Germany | K.H. Tully |
| Martini-Clinic | Hamburg | Germany | N. Atassi |
| Martini-Clinic | Hamburg | Germany | Stroelin |
| MCMCC - Warsaw | Warsaw | Poland | R. Sosnowski |
| Meander MC | Amersfoort | Netherlands | M. Schotman |
| Medisch Centrum Alkmaar | Alkmaar | Netherlands | J. Verlind |
| Medisch Centrum Alkmaar | Alkmaar | Netherlands | T. Roeleveld |
| Medisch Centrum Alkmaar | Alkmaar | Netherlands | S.D. Bos |
| Medisch Spectrum Twente | Enschede | Netherlands | E. Alleman |
| Medisch Spectrum Twente | Enschede | Netherlands | M. Asselman |
| Medisch Spectrum Twente | Enschede | Netherlands | B. Santerse |
| Medisch Spectrum Twente | Enschede | Netherlands | H. Leenknegt |
| Medisch Spectrum Twente | Enschede | Netherlands | M. Pit |
| Medisch Spectrum Twente | Enschede | Netherlands | S. Khoe |
| Mikkeli Hospital | Mikkeli | Finland | N. Hendolin |
| Miyazaki University Hospital | Miyazaki | Japan | N. Terada |
| Nagasaki University graduate school of medicine | Nagasaki | Japan | T. Hakariya |
| National Hospital Organization Kyoto Medical Cente | Kyoto | Japan | H. Okuno |
| Ng Teng Fong General Hospital | Singapore | Singapore | L.W. Tim |
| Niigata Cancer Center | Niigata | Japan | Y. Kitamura |
| Niigata University | Niigata | Japan | Y. Tomita |
| Osaka - Center for Adult Disease and Cancer | Osaka | Japan | M. Nakayama |
| Osaka University Graduate School of Medicine | Osaka | Japan | M. Uemura |
| Oulu University Hospital | Oulo | Finland | P. Hellström |
| Polycliniques les Bleuets | Reims | France | F. Staerman |
| Praxisgemeinschaft fur Onkologie und Urologie | Wilhelmshaven | Germany | G. Rodemer |
| Prince of Wales Hospital | Hong Kong | Hong Kong | C.F. Ng |
| Prince of Wales Hospital | Hong Kong | Hong Kong | P. Chiu |
| Prostatazentrum Elbe-Weser | Stade | Germany | C. Nitz |
| Prostatazentrum Elbe-Weser | Stade | Germany | S. Laabs |
| Reinier de Graaf Gasthuis | Delft | Netherlands |  |
| Rode Kruis Ziekenhuis | Beverwijk | Netherlands | N. Bosch |
| Ryukyu University Hospital | Okinawa | Japan | S. Saito |
| Saitama Cancer Center | Saitama | Japan | Y. Sakai |
| Sapporo Medical University Hospital | Sapporo | Japan | H. Kitamura |
| Sapporo Medical University Hospital | Sapporo | Japan | T. Tsukamoto |
| Seinäjoki Hospital | Seinäjoki | Finland | M. Leskinen |
| Shikoku Cancer Center | Matsuyama | Japan | K. Hashine |
| SIURO - ASST Bergamo Est | Bergamo | Italy | A. Paganelli |
| SIURO - Azienda Ospedaliera Careggi | Firenze | Italy | A. Lapini |
| SIURO - Azienda Sanitaria dell'Alto Adige | Merano | Italy | E. Trenti |
| SIURO - Azienda Sanitaria Giovanni XXIII | Bergamo | Italy | M. Roscigno |
| SIURO - Desenzano del Garda General Hospital | Desenzano | Italy | M. Tanello |
| SIURO - Fondazione IRCCS Istituto | Milan | Italy | F. Badenchini |
| SIURO - Fondazione IRCCS Istituto | Milan | Italy | T. Magnani |
| SIURO - Fondazione IRCCS Istituto | Milan | Italy | M.F. Alvisi |
| SIURO - Fondazione IRCCS Istituto | Milan | Italy | T. Rancati |
| SIURO - Istituto humanitas | Milan | Italy | L. Pasini |
| SIURO - Istituto regina elena | Rome | Italy | M. Gallucci |
| SIURO - Ospedale di Trento | Trento | Italy | O. Caffo |
| SIURO - Ospedale M. Bufalini | Cesena | Italy | G. Cicchetti |
| SIURO - Ospedale sant` anna | Como | Italy | G. Conti |
| SIURO - Ospedale umberto i | Nocera Inferiore | Italy | R. Sanseverino |
| SIURO - Policlinico di Bari | Bari | Italy | P. Ditonno |
| SIURO - Policlinico sant` orsola | Bologna | Italy | G. Martorana |
| SIURO - Università di Salerno | Salerno | Italy | V. Altieri |
| Slingeland Ziekenhuis | Doetinchem | Netherlands | A. Geboers |
| Spaarne Ziekenhuis | Hoofddorp | Netherlands | A. Noordzij |
| St. Agnes-Hospital | Bocholt | Germany | S. Tenbreul |
| St. Anna Ziekenhuis | Geldrop | Netherlands | A. Sonneveld |
| St. Antonius Ziekenhuis | Nieuwegein | Netherlands | P.L.M. Vijverberg |
| St. Franciscus Gasthuis | Rotterdam | Netherlands | I. van den Berg |
| St. Franciscus Gasthuis | Rotterdam | Netherlands | J. Blom |
| St. Franciscus Gasthuis | Rotterdam | Netherlands | E.R. Boevé |
| St. Franciscus Gasthuis | Rotterdam | Netherlands | R. Nooter |
| St. Franciscus Gasthuis | Rotterdam | Netherlands | J. Rietbergen |
| St. Franciscus Gasthuis | Rotterdam | Netherlands | S. de Vries |
| St. Franciscus Gasthuis | Rotterdam | Netherlands | I. van den Berg |
| St. Franciscus Gasthuis | Rotterdam | Netherlands | H. Wilkens |
| St. Jans Gasthuis | Weert | Netherlands | J.W. Langeveld |
| St. Marien Krankenhaus Ahaus-Vreden Gmbh | Ahaus | Germany | A. E.Treiyer |
| St. Savvas Hospital | Athens | Greece | T. Anagnostou |
| St.-Vincenz Krankenhaus | Datteln | Germany | M. Gillich |
| Stiftungsklinikum PROSELIS | Recklinghausen | Germany | J. Hanske |
| Tampere University Hospital | Tampere | Finland | T. Tammela |
| Teikyo University Chiba Medical Center | Ichihara | Japan | Y. Naya |
| Tergooiziekenhuizen | Hilversum | Netherlands | E. Bruijnes |
| Tergooiziekenhuizen | Hilversum | Netherlands | E. Hoogendijk |
| Tergooiziekenhuizen | Hilversum | Netherlands | F. van der Windt |
| Tergooiziekenhuizen | Hilversum | Netherlands | B. Zeijlemaker |
| Tochigi Cancer Center | Utsunomiya | Japan | K. Kawashima |
| Toho University Sakura Hospital | Sakura | Japan | N. Kamiya |
| Tohoku University Hospital | Sendai | Japan | K. Mitsuka |
| Tokai University Hospital | Isehara | Japan | T. Nomoto |
| Tokushima University Hospital | Tokushima | Japan | T. Fukumori |
| Tokyo Kosei Nenkin Hospital | Tokyo | Japan | K. Akakura |
| Tokyo Womens` Medical University | Tokyo | Japan | Y. Maeda |
| Tweesteden Ziekenhuis | Tilburg | Netherlands | B. Wijsman |
| UMC St. Radboud | Nijmegen | Netherlands | I. van Oort |
| UMC St. Radboud | Nijmegen | Netherlands | C.Hoeks |
| UMC/CMH | Utrecht | Netherlands | R. Lazarov |
| Univ. Hospital Muenster | Muenster | Netherlands | P. Papavassilis |
| Univ. Hospital Muenster | Muenster | Germany | A. Semjonow |
| Universitair Ziekenhuis Gent | Gent | Belgium | W. Oosterlinck |
| Université Jean Monnet | St Etienne | France | N. Mottet |
| University Hospital A Coruna | A Coruña | Spain | F.G. Veiga |
| University Hospital Malmö | Malmö | Sweden | A. Bjartell |
| University Hospital Príncipe de Asturias | Madrid | Japan | J.S. Uribe |
| University of Tokyo | Tokyo | Japan | H. Fukuhara |
| University of Tokyo | Tokyo | Japan | M. Nakamura |
| Urologia Marina Alta | Alicante | Spain | T. Keul |
| Urologische Gemeinschaftspraxis Buxtehude | Buxtehude | Germany |  |
| Urologische Klinik Sindelfingen | Sindelfingen | Germany | J. Trunk |
| USANZ - Ascot Hospital | Auckland | New Zealand | C. Hawke |
| USANZ - Auckland Hospital | Auckland | New Zealand | M. Rice |
| USANZ - Auckland Hospital, Private Practice | Auckland | New Zealand | M. Rice |
| USANZ - Austin and The Northern Hospital | Melbourne | Australie | D. Gyomber |
| USANZ - Austin Hospital | Melbourne | Australie | N. Lawrentschuk |
| USANZ - Ballarat Urology | Ballarat | Australie | L. Dodds |
| USANZ - Ballarat Urology | Ballarat | Australie | L. Johns-Putra |
| USANZ - Capital Urology Australia P/L | Canberra | Australie | H. Fan Chan |
| USANZ - Central Coast LHD | Gosford | Australie | F. Macneil |
| USANZ - Dr Mark Louie-Johnsun | Gosford | Australie | M. Louie-Johnsun |
| USANZ - Epworth Hospital | Melbourne | Australie | P. Anderson |
| USANZ - Geelong Urology | Geelong | Australie | K. Rantall |
| USANZ - Geelong Urology | Geelong | Australie | P. Kearns |
| USANZ - Metropolitan Urology | Melbourne | Australie | P. Ruljancich |
| USANZ - Nepean Urology Victoria | Mornington | Australie | A. Jayathillake |
| USANZ - Palmerston North Hospital | Palmerston North | New Zealand | C. Chemasle |
| USANZ - Palmerston North Hospital | Palmerston North | New Zealand | Q. King |
| USANZ - Perth Urology Clinic | Perth | Australie | S. La Bianca |
| USANZ - Peter MacCallum Cancer Centre | Melbourne | Australie | D. Murphy |
| USANZ - Private Practice Michael J Monsour | Launceston | Australie | M.J. Monsour |
| USANZ - Rockhampton Base Hospital | Rockhampton | Australie | A. Vega Vega |
| USANZ - St George Hospital | Sydney | Australie | C. Smiles |
| USANZ - St George Private Medical Centre Sydney | Sydney | Australie | D. Malouf |
| USANZ - The Alfred Hospital Melbourne | Melbourne | Australie | J. Grummet |
| USANZ - University of Sydney | Sydney | Australie | M. Patel |
| USANZ - University of Sydney | Sydney | Australie | V. Chalasani |
| USANZ - Urology Centre Port Macquarie | Port Macquarie | Australie | N. Awad |
| USANZ - Urology Centre Port Macquarie | Port Macquarie | Australie | P. Rashid |
| USANZ - Urology North | Launceston | Australie | S. Brough |
| USANZ - West Coast Urologists | Perth | Australie | A. Tan |
| Uwe Behrendt |  | Germany |  |
| Viecuri Medisch Centrum | Venlo | Netherlands | M. de Bruin-Titulaer |
| VUMC | Amsterdam | Netherlands | A. N. Vis |
| Wakayama Prefectural Medical University | Wakayama | Japan | I. Hara |
| Westfries Gasthuis | Hoorn | Netherlands | M. Leter |
| Yamagata University Hospital | Yamagata | Japan | N. Tsuchiya |
| Yamaguchi University Graduate School of Medicine | Yamaguchi | Japan | H. Matsuyama |
| Yamaguchi University Graduate School of Medicine | Yamaguchi | Japan | H. Matsumoto |
| Zaans Medisch Centrum | Zaandam | Netherlands | Soe Fung Kon Jin |
| Ziekenhuis Bernhoven | Veghel | Netherlands | A.Q.H.J. Niemer |
| Ziekenhuisgroep Twente | Hengelo | Netherlands | E.B. Cornel |
| Ziekenhuisgroep Twente | Hengelo | Netherlands | G. Molijn |
| Ziekenhuisgroep Twente | Hengelo | Netherlands | S.P. Stomps |
| Zuwe Hofpoort Woerden | Woerden | Netherlands | J. Beck |
| Zuwe Hofpoort Woerden | Woerden | Netherlands | S. van Selm |
| Zuyderland MC | Heerlen | Netherlands | T. Willems |
| Zuyderland MC | Heerlen | Netherlands | P. de Vries |
| Zuyderland MC | Heerlen | Netherlands | R. Bosshardt |
